# Perspective: A Conceptual Framework for Adaptive Personalized Nutrition Advice Systems (APNASs)

**DOI:** 10.1016/j.advnut.2023.06.009

**Published:** 2023-07-05

**Authors:** Britta Renner, Anette E. Buyken, Kurt Gedrich, Stefan Lorkowski, Bernhard Watzl, Jakob Linseisen, Hannelore Daniel, for the working group “Personalized Nutrition” of the German Nutrition Society, Johanna Conrad, Paola G. Ferrario, Christina Holzapfel, Michael Leitzmann, Margrit Richter, Marie-Christine Simon, Christian Sina, Jan Wirsam

**Affiliations:** 1 Department of Psychology and Centre for the Advanced Study of Collective Behavior, Psychological Assessment and Health Psychology, University of Konstanz, Konstanz, Germany; 2 Public Health Nutrition, Paderborn University, Paderborn, Germany; 3 ZIEL–Institute for Food and Health, Technical University of Munich, Freising, Germany; 4 Institute of Nutritional Sciences Friedrich Schiller University Jena, Jena, Germany, and Competence Cluster for Nutrition and Cardiovascular Health (nutriCARD) Halle-Jena-Leipzig, Germany; 5 Ex. Department of Physiology and Biochemistry of Nutrition, Max Rubner-Institut, Karlsruhe, Germany; 6 University Hospital Augsburg, University of Augsburg, Augsburg, Germany; 7 Institute for Medical Information Processing, Biometry, and Epidemiology, Ludwig-Maximilians-Universität München, Munich, Germany; 8 Ex. School of Life Sciences, Technical University of Munich, Freising, Germany

**Keywords:** personalized nutrition, public health, behavior change, food environment, dynamic system, advice, just-in-time adaptive intervention, framework, digital ecosystem

## Abstract

Nearly all approaches to personalized nutrition (PN) use information such as the gene variants of individuals to deliver advice that is more beneficial than a generic “1-size-fits-all” recommendation. Despite great enthusiasm and the increased availability of commercial services, thus far, scientific studies have only revealed small to negligible effects on the efficacy and effectiveness of personalized dietary recommendations, even when using genetic or other individual information. In addition, from a public health perspective, scholars are critical of PN because it primarily targets socially privileged groups rather than the general population, thereby potentially widening health inequality. Therefore, in this perspective, we propose to extend current PN approaches by creating adaptive personalized nutrition advice systems (APNASs) that are tailored to the type and timing of personalized advice for individual needs, capacities, and receptivity in real-life food environments. These systems encompass a broadening of current PN goals (i.e., what should be achieved) to incorporate “individual goal preferences” beyond currently advocated biomedical targets (e.g., making sustainable food choices). Moreover, they cover the “personalization processes of behavior change” by providing in situ, “just-in-time” information in real-life environments (how and when to change), which accounts for individual capacities and constraints (e.g., economic resources). Finally, they are concerned with a “participatory dialog between individuals and experts” (e.g., actual or virtual dieticians, nutritionists, and advisors) when setting goals and deriving measures of adaption. Within this framework, emerging digital nutrition ecosystems enable continuous, real-time monitoring, advice, and support in food environments from exposure to consumption. We present this vision of a novel PN framework along with scenarios and arguments that describe its potential to efficiently address individual and population needs and target groups that would benefit most from its implementation.

**Statements of significance**

This perspective provides a novel framework for personalized nutrition by creating adaptive personalized nutrition advice systems (APNASs).

## Introduction

For centuries, it has been commonly understood that individuals respond differently to food [1]. A growing number of empirical studies have demonstrated inter-individual variations in metabolic responses (e.g., blood glucose profiles) to food intake [2,3]. As information on common genetic variants becomes available, the concept of personalized nutrition (PN) has proliferated in academia and led to commercial applications following the presentation of the blueprint of the human genome. This scenario led to the most prominent and prevalent concepts of PN that refer to genetic differences based on single nucleotide polymorphisms or genetic risk scores associated with disease risks.

As the scientific field of PN constantly evolves, the definitions and concepts evolve and vary. Ordovas et al. [4] proposed that the overall goal of PN is “to preserve or increase health using genetic, phenotypic, medical, nutritional, and other relevant information about individuals to deliver more specific eating guidance to improve health or for delivery of nutritional products and services”. Similarly, Jinnette et al. [5] evaluated the effect of PN on changes in dietary intake based on 11 randomized control trials and defined PN “as an approach in which individual dietary intake, phenotypic information (e.g., anthropometric measurements and biomarkers of disease risk), and genetic information (e.g., single nucleotide polymorphisms) are used to design tailored nutrition advice” [6].

Over the past 20 y, research on PN and commercial services has used lifestyle information and biomedical parameters—primarily genotype or blood markers combined with established lifestyle-associated risks to individualize dietary advice [7]. More recently, genotype analysis (for review, see [8,9]) has been extended or replaced in commercial PN products by microbiome profiling [10]. In most PN approaches, the major argument is the appealing basic idea that personalized dietary advice results in better health outcomes. Although scholars have proposed in public media that the future of nutrition will be personalized, thus far, most programs are not based on empirical evidence and reach small consumer segments, generally health-conscious “early adopters” who are willing to pay for the service.

## Personalizing nutrition advice—setting better goals for what to eat

To personalize nutrition advice, current approaches focus predominantly on inter- and intra-individual differences in physiological or biological responses to food and nutrients [4,11] and their impact on physical health [12,13].

The basic assumptions behind such approaches are 2-fold. First, national food-based dietary guidelines, which provide generic 1-size-fits-all advice for a healthy choice, are considered “good but not good enough”. Second, deriving personalized dietary goals based on the physiological or biological responses to food intake is considered to refine the 1-size-fits-all advice, enabling precise advice toward optimal food choices. Accordingly, PN approaches typically entail a set of core steps, including *1*) selecting an optimal health outcome (in terms of disease prevention or physiological functions); *2*) collecting objective, valid, and precise measures; *3*) integrating available data; *4*) deriving data-based personalized goals (what should be achieved); *5*) communicating personalized goals; and *6*) monitoring progress toward achieving the goal.

Thus, PN approaches emphasize in-depth investigation of the individual physiological or biological make-up by collecting additional data apart from technological advancements and enhanced algorithms to aggregate increasingly large data volumes on biological systems. For example, various research initiatives intend to create integrated digital nutrition monitoring platforms to process and integrate data from various mobile sensors, such as electrochemical and motion sensors, visual images, and smart devices, using artificial intelligence (AI) and machine learning algorithms to derive personalized advice (e.g., see: [[14], [15], [16]]).

Although commercial PN applications and products frequently promise optimized health management for consumers, there is little scientific evidence that demonstrates an *add-on* individual health benefit. To date, scientific intervention studies predominantly evaluate the effect of PN advice based on genetics on improvement in the diet- and health-related indicators are limited and yield small effects and inconsistent results (for an overview, see [5,8,17]). Recent commercial PN approaches, which are based on gut microbiome analyses, are surging in value with an estimated market value of hundreds of millions of United States dollars by 2030 [18], in contrast with the lack of evidence for the add-on benefit of these concepts [10].

## Why personalizing nutrition goals may work–but because of other reasons than we think

PN approaches and applications are typically characterized by the assumption that providing detailed and frequent information about individual health indicators will lead to sustained behavior change and, ultimately, to better health [7]. In the field of risk communication, this method—the idea that making information available in detail and in increasing frequency—is called the “just-say-it approach” and frequently fails to change behavior [19]. However, on a relatively positive note, there are several reasons to assume that personalized goals may be met with increased acceptance compared to 1-size-fits-all goals, independent of the content of the advice provided by experts through an interplay of psychological mechanisms [5].

**FIGURE 1:**
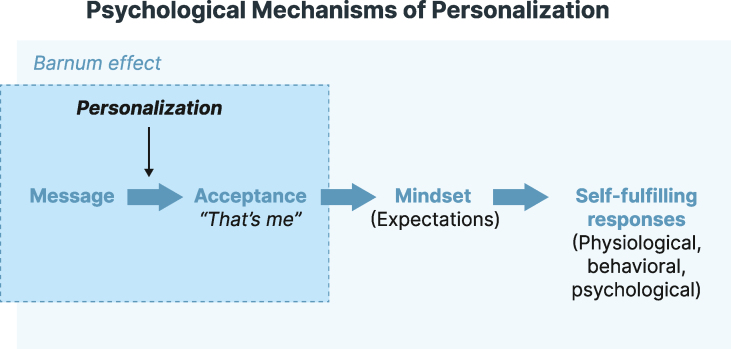
Mechanisms that underlie the value of personalization.

Regarding the perceived value of personalization, people typically hold positive views of their eating motives and behaviors compared with a peer of the same age and sex, that is, the average person; this is known as the “better-than-average” phenomenon [[20], [21], [22]]. In addition, providing personalized health advice may tap into the “Barnum effect”, which describes a psychological phenomenon where individuals demonstrate high levels of acceptance of descriptions of personal characteristics that are supposedly personalized to them; in fact, these characteristics are generic and equally apply to a broad range of people [23,24]. Therefore, people are more likely to accept advice that they believe has been personalized to them. Personalized generic communication is an age-old practice in fields such as marketing or health communication [25]. Consequently, personalized advice can induce a mental mindset with an array of expectancies, orienting people toward psychological, physiological, and behavioral responses in line with such expectations, which, in turn, create changes in a self-fulfilling manner (Figure 1). For example, merely learning about one’s genetic risk for a disease can alter the actual risk by making people more likely to exhibit expected changes in gene-related physiology, behavior, and subjective experience. Notably, Turnwald et al. [26] reported that the effects of (supposedly) personalized genetic risk information on outcomes were greater than the effects associated with actual genetic risk.

However, goal personalization may also entail unintended or even negative effects by setting overly ambitious goals, which may be met with failure if not adequately addressed in nutrition and lifestyle counseling. The psychology literature demonstrates that a larger discrepancy between the actual state and the aspired goal is likely to result in pessimistic *appraisals of means* (e.g., low self-efficacy and negative outcome expectancies; [27]) for most individuals. This notion results in discouraging setbacks or failures, which can lead to a negative downward spiral marked by behavioral disengagement [28,29]. Moreover, this scenario may contribute to the further widening of health disparities.

## Why personalizing nutrition might work–but not a priori for public health

**FIGURE 2:**
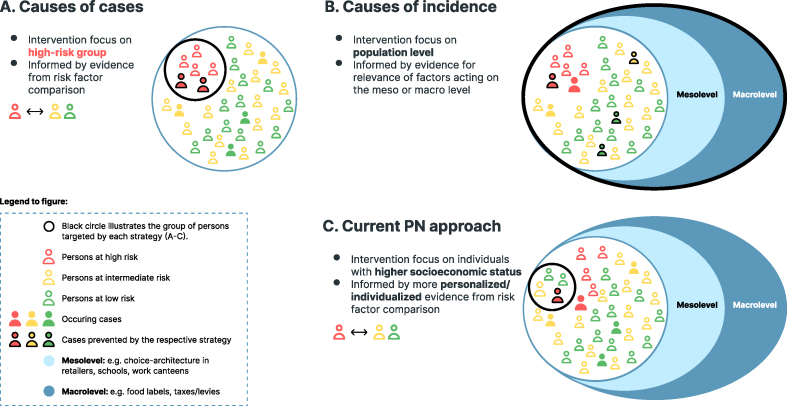
Causes of cases (A) and incidence (B), and current personalized nutrition concepts (C). PN, personalized nutrition.

Conceptually, PN can be viewed as an individualization of the classical high-risk approach because it is based on evidence from comparing risk factor profiles between individuals at high and those at low or intermediate risk of disease (Figure 2A). Hence, intervention tailored to these identified risk factors (causes of cases) can help prevent disease cases that are likely to occur in such vulnerable population groups. However, despite low baseline risks, the absolute number of diseases may be higher in population groups at low or intermediate risk (see bold icons in Figure 2A). In addition, the food environment exerts a powerful influence on food choices, hampering the success of intervention approaches in high-risk groups [30]. Scholars typically underestimate the extent of this influence [31] by not acknowledging that barriers introduced via the environment may impede healthy food choices. Specifically, these barriers may occur at the meso level, that is, limited access to opportunities for healthy food choices (e.g., retailer availability) and specific settings (e.g., schools or workplaces) [32]. Especially in less advantageous neighborhoods, the available and accessible food environment set by retailers and out-of-home services (e.g., takeaways and fast-food outlets) represents considerable barriers to implementing a healthy lifestyle [[33], [34], [35]]. Finally, the extent to which nutrition policies are implemented in a country influences the range and recipes of food products and choices available in the food environment at the macro level. The implementation encompasses reformulation policies to reduce sugar or salt content, prices, the degrees to which taxation levels or tax exemptions are implemented, and the presentation of products, that is, whether or not front-of-package labels are in place [30]. Comparisons of traditional risk factors cannot help estimate the relevance of these factors to the incidence of diseases because they represent ubiquitous potential drivers of diseases [36]. Instead, approaches for public health nutrition focus on implementing healthier, fairer, and more sustainable food environments that are expected to provide benefits to populations as a whole (Figure 2B). Hence, they are likely to be cost-effective and beneficial to social equity [[37], [38], [39]]. The currently advocated PN approaches might result in successful individual changes; however, such individual improvements are unlikely to entail commonly proposed benefits on a public health scale [4]. Concerns not only arise because PN approaches overlook the ubiquitous risk factors that act on the meso and macro levels but also because of their nature, which primarily addresses individuals with considerable resources and appropriate access to these offers which may not be at high risk for disease (Figure 2C). In turn, the social inequality promoted by such endeavors is of concern [40], given that social inequality is, a priori*,* a major cause of many diseases across populations [41].

## Why setting better or personalized goals for what to eat might not be enough—the action gap

Nowadays, eating healthily and avoiding weight gain or losing weight are collective desires. In the United States, the proportion of people who intended to lose weight increased from 34% in 1999–2000 to 42% in 2015–2016 [42]. Moreover, awareness of the importance of body weight and lifestyle factors for health is generally high and frequently overestimated [43]. However, people often fail to accomplish the desired dietary goals despite major public interest and a collective effort to eat healthily (e.g., see [44]).

**FIGURE 3:**
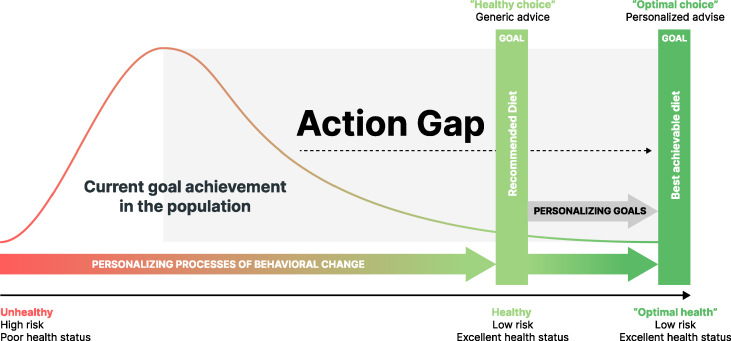
Schematics of the action gap between individual dietary behaviors and goals as defined by national agencies or with personalized approaches and the required behavioral changes.

If the action gap could be bridged for more people, enabling them to achieve their desired dietary goals, the generic 1-size-fits-all advice for healthy choices would indicate an enormous positive shift from the public health point of view (Figure 3). However, creating perfectly personalized nutritional goals cannot overcome the main challenges that most individuals and societies face. In other words, although optimizing the already good generic nutritional goals may provide additional health benefits, the more pressing issue is empowering people to adapt their daily behavior to reach their desired goals. Notably, approaches to address the action gap are frequently 1-size-fits-all approaches as well (i.e., the just-say-it approach) and do not consider the dynamic, multi-factorial, and idiosyncratic nature of nutritional behavior [45,46]. Moreover, from the perspective of public health, individual strategies that mitigate individual causes of diseases do not address the causes of incidence at the population level and may thus exert only a small effect at the population level, as previously discussed [40].

Figure 3 shows that most approaches to PN are focused on sharpening biomedical goals without addressing the action gap and do not question how consumers and patients can implement the given advice in daily life. In other words, the personalization of nutrition advice is related not only to deriving personalized goals (what), but also to personalizing the process of behavioral change (how).

## Why 1-size-fits-all behavior change approaches are not enough—the dynamics and multi-functionality of individual eating behaviors

Food is ubiquitous in everyday life, and eating appears to be a simple activity; however, it is a complex behavioral process involving ≤200 decisions/d [47]. People need to decide what, how much, where, when, how, and with whom they eat or do not eat. Thus, eating behavior is a repeated occurrence behavior performed over the entire lifespan in different contexts and is highly dynamic because it varies daily at the individual level ([45]; see also [[48], [49], [50]]).

Recent in situ and *in-the-moment* high-resolution behavior assessments revealed *idiosyncratic* behavioral signatures with a high variability of a particular eating behavior and its underlying processes not only between but also within individuals. For example, (near) real-time tracking of eating events along with 15 motives for 8 consecutive days in real-life exhibited marked inter-individual differences in intra-individual eating motive profiles [51]. Similarly, palatability ratings seemingly varied more from day to day for the same individual than between individuals [52]. Moreover, individuals’ actual activities and experiences in the moment, at certain times and in situ within certain contexts differ substantially from their remembered experiences (the memory–experience gap; e.g., [53]). Mobile in-the-moment and in situ assessments also revealed marked differences between experienced and remembered eating events [54].

Eating behavior is multi-factorial and ranges from physiological (e.g., hunger) and psychological (e.g., positive or negative emotional states) to economic (e.g., income) and social reasons such as commensality and norms [[55], [56], [57], [58], [59]]. Hence, in addition to hunger and taste, other compelling reasons exist for what, how much, and how we eat, which are part of normal human eating behaviors. For example, studies in Brazil, Germany, India, the United States, and other countries consistently yielded 15 eating motives [60,61]. Thus, although food cultures and eating practices differ widely across geographical regions and ethnic groups, people across food environments share a set of basic eating functions [57,61]. For example, most people prefer not to eat alone and consider such a meal to be “unreal” [62,63]. Empirical studies suggest that commensality, or social dining, exerts beneficial effects on nutritional status, well-being, and social cohesion (see [64] and [65] for a review). Similarly, environmental and sustainability concerns are becoming increasingly important factors in shaping individual choices (e.g., see [[66], [67], [68]]). Accordingly, we propose that incorporating individual goal preferences (e.g., pleasure, commensality, and making sustainable dietary choices) beyond the currently advocated biomedical targets is a core aspect of future PN solutions.

**FIGURE 4:**
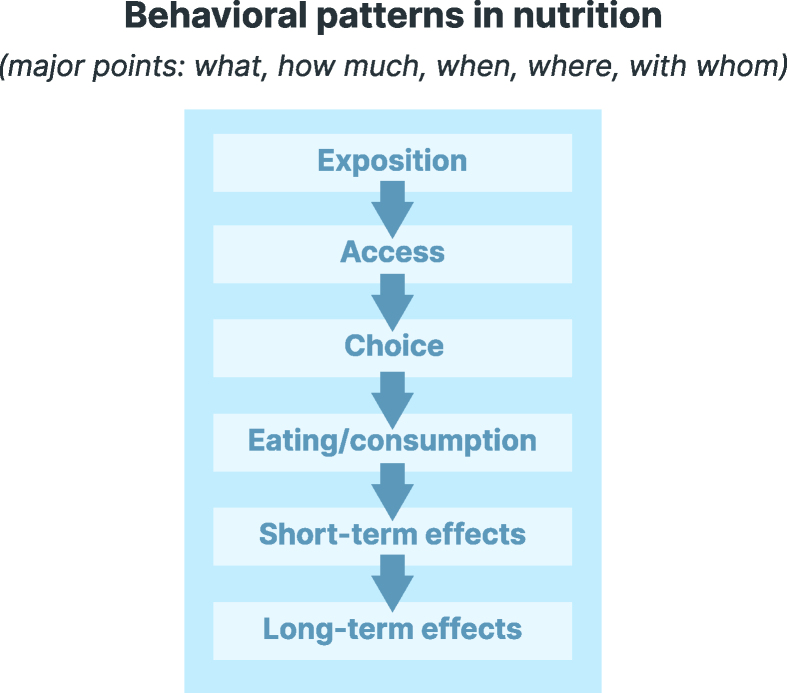
Event sequence in the food environment [31,70].

Eating behavior depends on conscious, reflected decisions and a combination of available options, habits, and influences, of which individuals may even be unaware (see also section “Why personalizing nutrition might work—but not for public health”). The “food environment,” which forms the context of nutritional behavior (see [31,59,69,70]), shapes eating behavior decisively. Given that the food environment is the sum of all environmental factors influencing nutritional behavior, eating is a result not only of decisions made in moments of concrete consumption but also of a behavioral process that spans phases of exposure (i.e., what people see and perceive in their environment every day shapes the concept of social norms), access (i.e., which foods are physically accessible and socially acceptable to people), choice (i.e., which products are purchased or selected), and, eventually, consumption (i.e., which foods, meals, or snacks are eaten; Figure 4).

For example, frequent exposure to specific physical food environments such as fast-food outlets is associated with unhealthy diets and high rates of obesity (for review, see [71]). A large-scale tracking study with >1.1 million participants from the United States and 2.3 billion food entries found that high access to grocery stores, low access to fast food, a high income, and college education were all independently associated with high levels of consumption of fresh fruits and vegetables, low levels of consumption of fast food and soda, and less likelihood of being overweight or obese [35]. Moreover, the social environment exerts a pervasive and powerful influence on what and how much people eat (for an overview, see [72]). At the societal macro level, for instance, mealtimes shape collective eating behaviors and social lives as social norms with pronounced differences between countries and eating cultures [73]. Therefore, incorporating environmental context into PN advice (i.e., where and when) may be a promising avenue. The first evidence for this notion is provided by a recent study that displayed a higher acceptance rate of PN advice at lunch than breakfast or dinner [74].

Taken together, the generic 1-size-fits-all behavior change advice does not adequately address the multi-functionality and high inter- and intra-variability of eating across different food environments [75] and lacks the required emphasis on behavior changes from a systems perspective (see [46] for a discussion of behavior change from the systems perspective). To facilitate behavior change and leverage the potential of PN, the multi-functionality of eating behavior beyond physical health needs to be considered; PN advice needs to be adapted to dynamic individual behavioral signatures in situ and in time.

## Why we need to combine different ways of personalization—static and adaptive approaches to change behavior

Although scholars acknowledge the multi-factorial nature of eating behavior and environmental influence, these factors and their effects are frequently conceptualized as uniform or static (for an overview, see [76]). For instance, stress is frequently viewed as a major environmental factor contributing to overeating and obesity (for a review, see [77,78]). Assuming this relationship is static and uniform, it would not require personalizing the advice provided.

However, marked differences exist in response to stress episodes; stress-hyperphagic individuals eat more, whereas stress-hypophagic individuals eat less in response to stress episodes [[79], [80], [81]]. In contrast to the generic approach, the behavior change approach could be personalized by matching it to particular groups based on relatively stable personal characteristics (i.e., building self-regulation capacity within stress-hyperphagic individuals). In relation to PN, Dijksterhuis et al. [82] identified 4 psychosocial types of consumers who differed according to advice preference and need.

**FIGURE 5:**
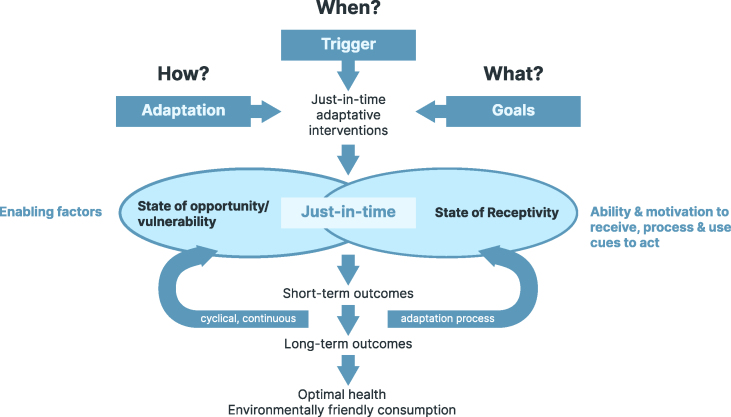
Framework for just-in-time adaptive interventions that lead to favorable outcomes (adapted from [83]).

Stress responses and emotional eating vary not only between individuals but also within them depending on social and physical environments (e.g., see [48]); thus, adapting interventions to the changing state of individuals within different environments may be more effective. A framework for adaptive behavior change interventions is just-in-time adaptive interventions (JITAIs) [83]. By providing just-in-the-moment advice, JITAIs intend to personalize behavior change approaches when the person is at risk of engaging in a negative behavior (state of vulnerability) and/or the person gains an opportunity to engage in a positive behavior (state of opportunity; Figure 5). Moreover, advice is provided when the person is receptive to support and displays the ability and motivation to use the advice. The personalization of behavior change interventions in the context of JITAIs is a dynamic process; ideally, the provision of advice is adjusted to the changing needs of individuals (e.g., changing the timing or type of advice) during the course of the intervention [83,84]. Thus, JITAIs are continuously *tuned* to the evolving needs of individuals by providing the appropriate component with the most effective amount at the right time by adapting to the changing internal and contextual states of individuals in real-time and adjusting immediate goals to support behavior change [76]. Hence, to adapt the advice, the questions—for what, when, where, and how—for which advice can be provided need to be addressed repeatedly.

The widespread daily use of mobile technologies, such as smartphones, sensors, and other wearable devices, increases the feasibility of in-time and in situ interventions for personalized behavior change. Various mobile programs (e.g., Lose it!, Meallogger, WeightWatchers, and Noom) use psychological and behavior change techniques (BCTs) to target nutrition behaviors and nutrition-related health outcomes. A meta-analysis of data from 41 studies including 30 different applications (apps) (18 of these were fully mobile programs) demonstrated that app-based mobile interventions, which delivered behavior change interventions in situ and/or in the moment, improved nutrition behaviors and related health outcomes significantly (e.g., BMI (in kg/m^2^), weight, and blood parameters) in a wide range of study settings with patients and generally healthy study populations [85]. The common building blocks of these interventions were four of the 16 major clusters of psychological BCTs, namely, goal setting, feedback and self-monitoring, information, and social support provision, which coincide with successful conventional individual and group interventions. Although the initial results are promising, the research on JITAIs remains in the early stages [76].

## What could PN look like in the future? A conceptual framework for adaptive PN advice systems

From this perspective, the current argument provides evidence that current PN approaches need to transcend the predominant focus on inter- and intra-individual differences in physiological or biological responses to food and nutrients [4,11] and their impact on physical health [12,13].

**FIGURE 6:**
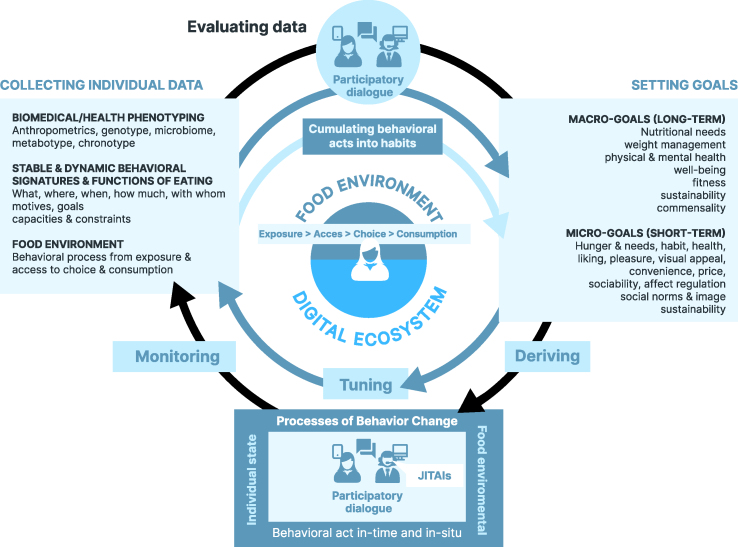
Adaptive personalized nutrition advice systems and their key features. JITAI, just-in-time adaptive intervention.

The most robust approaches to personalization will include biological and psychological-behavioral systems—biology to understand an individual’s needs and psychological and BCTs to take appropriate actions to address biological needs and meet user-defined goals (see also [4,13,86]). Our vision is for adaptive PN advice systems (APNASs) that focus on setting individual goals and tailoring adaptive behavior change processes to accommodate individual needs, capacities, and receptivity in real-life food environments. This encompasses a broadening of the current PN goals (what should be achieved) to incorporate individual goal preferences beyond the currently advocated biomedical targets (e.g., making sustainable dietary choices) (see also [13,86] for a related view). It includes the personalization processes of behavioral change by providing in situ and just-in-time information in real-life food environments (how and when to change), considering individual capacities and constraints across contexts (e.g., available behavioral options and economic resources). Finally, it is based on a “participatory dialog between individuals and experts” (e.g., an actual or virtual dietician, nutritionist, or advisor) when setting goals and deriving behavior change processes. These APNASs build on the collection of individual data to select and prioritize individualized short- and long-term goals and possibilities for changing behaviors with the following core features (Figure 6).

### Collecting individual data

Instead of focusing on in-depth genetic and metabolic phenotyping, in-depth profiling of individual behavioral signatures and food environments should be conducted, beginning with relatively stable individual and environmental characteristics to derive individual goal preferences and the initial leverage points for the processes of behavioral change. This step will be completed with individual data collected in time and in situ to increasingly “tune” JITAIs based on individual data, enabling the processes of behavioral change [76]. High-resolution, multidimensional behavior assessment through mobile devices “in the wild” offer great potential to assess idiosyncratic behavioral signatures in situ and in real-time without relying on human memory or input. Similar to physiological sensing, as in behavioral sensing, machine learning is increasingly used for pattern recognition and prediction (e.g., for identifying situations and timing interventions to trigger a personalized intervention before an eating occasion or in a specific environment).

### Setting goals

Current PN goals need to be broadened, notably to incorporate individual goal preferences beyond the currently advocated biomedical targets. This could include long-term goals concerning mental health, well-being, fitness, or enjoyment. Importantly, consumers may exhibit strong preferences toward other values, such as increased sustainability in food consumption (e.g., greater social, environmental, or animal welfare comparability). Typically, goals should fit the basic functions of normal human eating behaviors, such as social traditions and commensality. Eating motives at the moment (“micro-goals”) may differ considerably from long-term ones (“macro-goals”) because of individual states and environments, which require dynamic adjustments to address conflicting goals and create synergies. Thus, the selection and prioritization of macro- and micro-goals should be matched to individual preference structures and capacities.

### Enabling processes of behavior change

**FIGURE 7:**
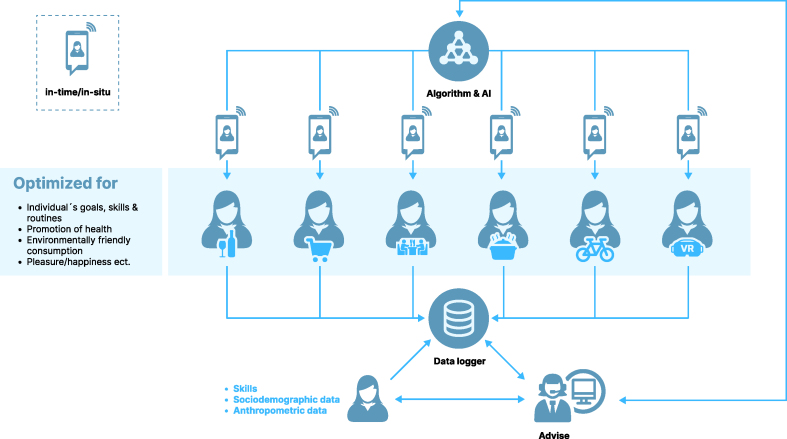
Example of an adaptive personalized nutrition advice system (APNAS) that addresses behavioral acts in time and in situ by providing a vision of the new quality in a built virtual reality. AI, artificial intelligence.

At the heart of the model is the proposal that phenotyping does not primarily require additional personalization; rather, the processes of behavioral change require personalization. Within this premise, the entire program for behavioral change, developed through a participatory process, must enable advice and JITAIs adapted to the changing internal and contextual states in situ and in-time per individual. Moreover, boosting individual capacities by prioritizing behavioral change processes enabling individuals to act in the moment and in situ (behavioral act) is important. How people may act in the moment may differ considerably for each case because of the individual states and food environments. To cumulate behavioral acts into habits and long-term behavioral patterns, dynamic selection and prioritization of targeted behavioral acts matched to individual preference structures and capacities are required (Figure 7). These processes need to be continuously tuned to the individuals’ changing internal and contextual states.

### Participatory dialog between individuals and experts

During the process of collecting data, evaluating options, setting goals, and deriving processes of behavioral change, individuals need to be actively involved at all levels of decision-making. To date, participation is frequently only exercised at the level of consultation. Importantly, this participatory dialog needs to account not only for the capacities but also the constraints perceived by individuals. Thus, the strategy must be tailored to the person rather than fitting the person to the strategy. Digital integrative platforms offer possibilities of actively involving users on all levels of decision-making via (actual or virtual) participatory interaction. This has implications for the expert teams, virtual coaches, and advice programs which—in addition to nutritional knowledge—require knowledge of behavior change and digital systems.

Although APNASs project an image of the future, numerous commercial applications and services (e.g., out-of-home food services and marketing) previously collected and used intensive data from digital environments to influence decisions and behaviors. This notion demonstrates the tremendous impact and potential of adaptive systems, which can be used to tailor the type and timing of personalized advice to accommodate individual needs, capacity, and receptivity in real-life environments (e.g., [[87], [88], [89]]). For example, modern machine learning systems such as personalized recommender systems learn user preferences to deliver recommendations that change online behavior (a prominent example is Netflix; see [90,91]) and use personalized, context-aware re-ranking algorithms that are integrated with the Internet of Things to influence behavior. These applications and services are becoming increasingly automated, interactive, and personalized because they use sensors and other user data to tailor interventions without the need for inputs from experts or professionals [92]. An important feature is that they aim to create JITAIs adapted to the capacities of individual users within specific contexts. New technologies such as conversational agents (also known as chatbots) facilitate this development. These computer systems imitate human conversation using text or spoken language and offer personalized human-like interactions (e.g., [93]). An example is an AI-powered virtual human that answers questions about baking cookies, which was recently introduced by one of the largest food producers worldwide. Moreover, developments such as ChatGPT or DALL E 2 by OpenAI show that adaptive digital ecosystems have reached a new scale at an enormous pace [94]. Thus, the potential for integrating different sensors, services, and AI is huge. Depending on the question and type of advice, there are already concepts or ideas, for example, the kitchen of the future or services using AI to create new solutions, such as making new recipes from scratch in situ and in time and connecting these with retail services and kitchen applications.

Although a certain amount of evidence exists on the potentially harmful effects of these new digital ecosystems and the over-reliance on AI approaches ([88,89]; see also “dark patterns” in [95,96]), utilizing them in creating APNASs by personalizing the goals and processes of the behavioral change via real-time monitoring, advice, and support in food environments from exposure to consumption is valuable. For example, as part of an APNAS digital ecosystem (Figure 7), users received suggestions for an easy recipe, which they could then prepare on the spot using currently available ingredients. Thus, the vision is to address consumer choices by adapting the nutrition advice in situ and in time according to individual goal preferences (e.g., food preferences, sustainability concerns, and commensality), individual capacities (e.g., cooking abilities), and constraints (e.g., available ingredients and kitchen appliances). Through users’ responses, the underlying AI constantly learns and adapts to individual preferences, abilities, and constraints. By interconnecting technologies and services with built-in application programming interfaces, products and discounts from nearby retailers are offered. Moreover, retailers and kitchen appliance companies are part of the digital ecosystem; they can tailor their products and services according to user profiles. Thus, access and the choice architecture of the environment are tailored to individual consumers by interlinking out-of- and in-home domains, creating JITAIs that can adapt to the capacities of individual users within specific contexts.

Creating an APNAS digital ecosystem entails various challenges. The adoption and use of digital platforms and mobile services are influenced by multiple factors within individuals (e.g., motivational readiness and goals) and across applications or platforms (e.g., usability, technical issues, and costs) and the environment (e.g., social influence or context [97,98]; see also the affect–integration–motivation and attention–context–translation AIM-ACT model for in-the-moment engagement in digital interventions by [99]). APNASs address these aspects by incorporating individual goal preferences and personalizing processes of behavior change that consider the constraints across contexts. Empirical evidence supports this notion: first, various mobile nutrition apps affect BMI, body weight, and blood parameters favorably [85]; second, suitable prompts lead to low rates of missing data [[100], [101], [102]], and customizable features increase user engagement [103]; and third, customized mobile health programs show high engagement rates over months in large groups including community-dwelling middle-aged and older adults (see for example, the Stop Diabetes intervention study in Finland [104]). From a public health perspective, the major concern is that these digital ecosystems will increase social inequality by primarily targeting individuals with or without minor risks but with considerable resources or by using dark patterns to deceive vulnerable users. Although technology literacy is related to age, digital experience, literacy, education, cultural background, and residency, the evidence for a digital divide in the uptake, engagement, and effectiveness of mobile interventions for the promotion of weight-related behaviors is inconsistent and inconclusive [105]. Moreover, food environments such as food retail and out-of-home services and food-related behaviors are increasingly shaped by digitalization across socioeconomic groups—a multi-country study showed differences in the proportion of food delivery apps users from low- and high-income groups between countries—however, overall, food delivery apps are accessed by all groups to a high degree, regardless of income [106]. Notably, a recent start-up in the United States implemented a digital platform to address food insecurity. Registered users automatically receive daily information via text messages about the food options available to them from grocery stores and food providers (e.g., takeaways or restaurants) in situ and in time. Thus, *tuning* services and advice tailored to individual behavioral signatures, preferences, capabilities, and environmental characteristics has the potential to reach persons from all socioeconomic backgrounds.

Our vision of building an APNAS digital ecosystem places users at the center, focusing on individual goals and preferences to find the best leverage points for behavioral change in various food environments. By adapting to individual preferences and capacities within environments, the reasons and incentives to use APNASs are more diverse, ranging from macro to micro-goals across domains, compared to the PN approaches that are currently employed, which focus nearly exclusively on biomedical or health goals. Hence, health can be the core motivation for individuals to change but not necessarily. However, APNAS digital ecosystems need to be affordable, accessible, secure, and trustworthy, with an open data infrastructure for further services to take a step forward in addressing the action gap and causes of cases across populations. This vision represents substantial technical challenges as well as potential conflicts with the interests of stakeholders. This is because data and intelligent algorithms are frequently an inherent part of business models. A potential future perspective is that APNAS digital ecosystems and their technical infrastructures are part of a public sphere and services [107] in empowered data societies [108]. Currently, it would be challenging to implement an APNAS digital ecosystem practically; however, we are convinced that the technical and legal aspects of such an implementation are relevant, as is the discussion regarding the types of digital ecosystems we want to create.

In conclusion, this study reveals the limitations in the concepts of PN, which was introduced ∼20 y ago, in terms of their effectiveness in changing dietary or lifestyle behaviors. Moreover, these approaches target privileged groups of consumers, who are typically well-educated, health-conscious, and can afford such services; thus, they provide only marginal benefits to wider public health. Thus, this scenario calls for new PN approaches by increasing the focus on individual preferences, capacities, and goals. These approaches may address the 1-dimensional health perspective of the current concepts by including, for example, environmentally friendly consumption. The study proposes the utilization of BCTs combined with the tools of digital ecosystems for targeted dynamic and adaptive intervention systems that support in-time and in situ decision-making on consuming and preparing food. Moreover, other parameters such as physical activity or social interactions and the provision of pleasure, overall happiness, and well-being should be considered. This comprehensive approach requires constant interaction between multiple advisors or advice systems, which may be avatars or (user-defined) chatbots. Using the new tools in the digital world and self-learning systems, this approach can be targeted to any social group; retailers, health insurance organizations, or public bodies (or combinations thereof) may adopt its skills and capacities to provide added value for a better life and improved public health in the “1-health dimension” of the food, diet, and health sequelae.

## Acknowledgments

We thank the members of the Working Group “Personalized Nutrition” of the German Nutrition Society–Johanna Conrad, Paola G Ferrario, Christina Holzapfel, Michael Leitzmann, Margrit Richter, Marie-Christine Simon, Christian Sina, and Jan Wirsam.

### Author contributions

The authors’ responsibilities were as follows– BR: prepared the first draft with critical input from AEB, KG, JL, and HD; All authors contributed to framework development and critically edited the manuscript, and all authors: read and approved the final manuscript.

### Conflict of Interest

All other authors report no conflicts of interest.

### Funding

BR leads the “Collective Appetite” project within the CASCB (Centre for the Advanced Study of Collective Behavior), funded by the German Research Foundation (DFG) under Germany’s Excellence Strategy (grant number: EXC 2117–422037984) and the “SMARTACT” project (grant number: 01EL1820A) funded by the German Federal Ministry of Education and Research [Bundesministerium für Bildung und Forschung, (BMBF)]. AEB receives funding for the Joint Programming Initiative “A healthy Diet for a Healthy Life” and ERA-NET Cofund HDHL INTIMIC METADIS project CarbHealth (BMBF grant number: 01EA1908A) and the ChroNu study funded by the German Research Foundation (DFG) (grant number: BU 18073-2). KG is a partner in the Food Nutrition Security-Cloud research consortium funded by the European Union’s Horizon 2020 Research and Innovation program (grant agreement number: 863059) and the Third Bavarian Nutrition Survey (A/19/15) funded by the Bavarian State Ministry of Nutrition, Agriculture and Forestry [Bayerisches StMELF (Staatsministerium fpr Ernährung, Landwirtschaft und Forsten)]. Furthermore, KG leads the German arm of the Food, and You study with funding from the Ecole Polytechnique Fédérale de Lausanne. SL is the coordinator of the Competence Cluster for Nutrition and Cardiovascular Health (nutriCARD) Halle-Jena-Leipzig (grant numbers: 01EA1411A and 01EA1808A) funded by the German Federal Ministry of Education and Research (BMBF). BW is currently the president of the German Nutrition Society. JL received funding for the Joint Programming Initiative “A Healthy Diet for a Healthy Life” project DIMENSION (BMBF, grant number: 01EA1902B), for a project in the context of the Nutrition Competence Network ENABLE (BMBF, grant number: 01EA1807E), for the project RIDE-PPI funded by the Innovation Fund (G-BA, grant number: 01VSF18013), and for the project BVS III (Bavarian Ministry for Nutrition, Agriculture, and Forestry, grant number: A/19/15). The German Nutrition Society is a nonprofit science organization that receives funding from the German Federal Ministry of Food and Agriculture.
